# Concentration of EPA and DHA from Refined Salmon Oil by Optimizing the Urea–Fatty Acid Adduction Reaction Conditions Using Response Surface Methodology

**DOI:** 10.3390/molecules24091642

**Published:** 2019-04-26

**Authors:** Gretel Dovale-Rosabal, Alicia Rodríguez, Elyzabeth Contreras, Jaime Ortiz-Viedma, Marlys Muñoz, Marcos Trigo, Santiago P. Aubourg, Alejandra Espinosa

**Affiliations:** 1 Department of Food Science and Chemical Technology, Faculty of Chemical and Pharmaceutical Sciences, Santos Dumont 964, University of Chile, Santiago 8380000, Chile; gretel.dovale@ug.uchile.cl (G.D.-R); ely_tcn@hotmail.com (E.C.); marlys_dayane@hotmail.com (M.M.); 2 Department of Food Technology, Marine Research Institute (CSIC), Eduardo Cabello, 6, 36208 Vigo, Spain; mtrigo@iim.csic.es; 3 Department of Medical Technology, School of Medicine, University of Chile, Santiago 8380000, Chile; bespinosa@med.uchile.cl

**Keywords:** refined commercial salmon oil, *n*-3 long-chain polyunsaturated fatty acids (*n*-3 LCPUFAs) concentration, EPA, DHA, EPA+DHA, total FA yield, process variable maximization, response surface methodology (RSM), multiple response optimization, desirability function

## Abstract

This research focused on obtaining eicosapentaenoic acid (EPA, 20:5 *n*-3) and docosahexaenoic acid (DHA, 22:6 *n*-3) (EPA+DHA) concentrates from refined commercial salmon oil (RCSO). Independent variables of the complexation process were optimized by means of the application of response surface methodology (RSM) in order to obtain the maximum content of such fatty acids (FAs). As a result of employing the optimized conditions for all the variables (6.0, urea:FA content ratio; −18.0 °C, crystallization temperature; 14.80 h, crystallization time; 500 rpm, stirring speed), high contents of EPA and DHA could be obtained from RCSO, achieving increases of 4.1 and 7.9 times in the concentrate, with values of 31.20 and 49.31 g/100 g total FA, respectively. Furthermore, a 5.8-time increase was observed for the EPA + DHA content, which increased from 13.78 to 80.51 g/100 g total FA. It is concluded that RCSO can be transformed into a profitable source of EPA and DHA (EPA+DHA), thus leading to a product with higher commercial value.

## 1. Introduction

In recent years, it has been recognized that the consumption of eicosapentaenoic acid (EPA) is associated with a low prevalence of coronary, circulatory, and inflammatory diseases [1,2,3,4]. Furthermore, docosahexaenoic acid (DHA) has been associated with fetal development, the prevention of neurodegenerative diseases, and the correct functioning of the nervous system and visual organs in the fetus [5,6,7,8,9,10]. According to the Food and Agriculture Organization/World Health Organization (FAO/WHO) [1], the recommended intake of EPA+DHA is at least 250 mg/day for adult males and non-pregnant/non-lactating adult females. Interestingly, the optimal brain development of children would need a 150 mg/day diet of such fatty acids. EPA+DHA concentrates may be produced by various methods, such as supercritical fluid chromatography, supercritical fluid fractionation, molecular distillation, silver complexation, enzymatic methods, and urea complexation [11,12]. Among them, complexation with the urea can be considered as the most efficient method, since polyunsaturated fatty acids (PUFAs) may be separated from saturated and monounsaturated ones by means of an economic process at low temperature [13,14,15,16,17,18]. The present research was focused on the employment of refined commercial salmon oil (RCSO) as a profitable source of EPA+DHA concentrates, which in time could lead to a product of higher commercial value. For it, independent variables of the urea adduction reaction conditions (urea:FA content ratio, crystallization time and temperature, and crystallization stirring speed) were optimized by response surface methodology (RSM) in order to achieve the maximum content of EPA, DHA and EPA+DHA. Additionally, the quality of the starting salmon oil was determined and evaluated.

## 2. Results

### 2.1. Characterization of the Initial Refined Commercial Salmon Oil

Results for the composition of RCSO reported that the most abundant fatty acids were 18:1 9c (29.61%), 18:2 9c, 12c (16.69%), and 16:0 (13.74 %) followed by EPA (7.53%) and DHA (6.25%) (g/100 g total FA). The total value of saturated fatty acids (SFAs) was 21.28%, which was composed mainly of palmitic, stearic, and myristic acids (Table 1). Special interest is the confirmation of the absence of phytanic acid within the lipid composition of RCSO. This fatty acid has been linked to neurological disorders in some people, but it is also associated with the prevention of metabolic syndrome or type 2 diabetes [19].

Values obtained for the oxidative stability of RCSO were: peroxide value (PV) = 5.23 ± 0.05 meq. active oxygen kg^−1^ oil; p-anisidine value (pAV) = 6.84 ± 0.46; and total oxidation value (TOTOX) = 17.30 ± 0.40 and free fatty acids (FFA) = 0.30 ± 0.01 g oleic acid/100 g oil. Previous studies performed on refined salmon oil samples [18] indicated average values of PV= 3.54 ± 0.16 meq active oxygen kg^−1^ oil; p-anisidine value (pAV) = 5.14 ± 1.02 and FFA= 0.23 ± 0.00 g oleic acid/100 g oil. and at and 12.22 ± 1.34, respectively.

### 2.2. Effect of Process Variables on Total FA Yield, EPA Contents, and DHA Contents of RCSO Concentrate

#### 2.2.1. Refined Commercial Salmon Oil Concentrate

According to the experimental design, showed in Table 2, 28 assays were performed to obtain different refined commercial salmon oil concentrates. This table reports the experimental values obtained for the different response variables: R_1_ (total FA yield; g FA in the non-urea complexing fraction/100 g initial saponified oil FA), R_2_ (EPA content; g/100 g total FA), and R_3_ (DHA content; g/100 g total FA) of RCSO concentrate. Table 2 also includes the predicted values for the described corresponding variables, whereas the experimental values were replaced by the application of the model (R1’, R2’, and R3’ values, respectively). As a result, all the independent variables (A: urea/FA content ratio, *w*/*w*; B: crystallization temperature, °C; C: crystallization time, h; D: stirring speed, rpm) significantly affected (*p* < 0.05) the response variables during the urea complexation process. On the other hand, the enrichment of EPA and DHA in concentrates varied inversely according to total FA yield, obtaining correlation coefficient values (r) of −0.7756 and −0.7185, respectively.

#### 2.2.2. Effect of Process Variables on EPA, DHA, and EPA+DHA Content and Total FA Yield: Pareto Charts and RSM Analysis

**Figure 1:**
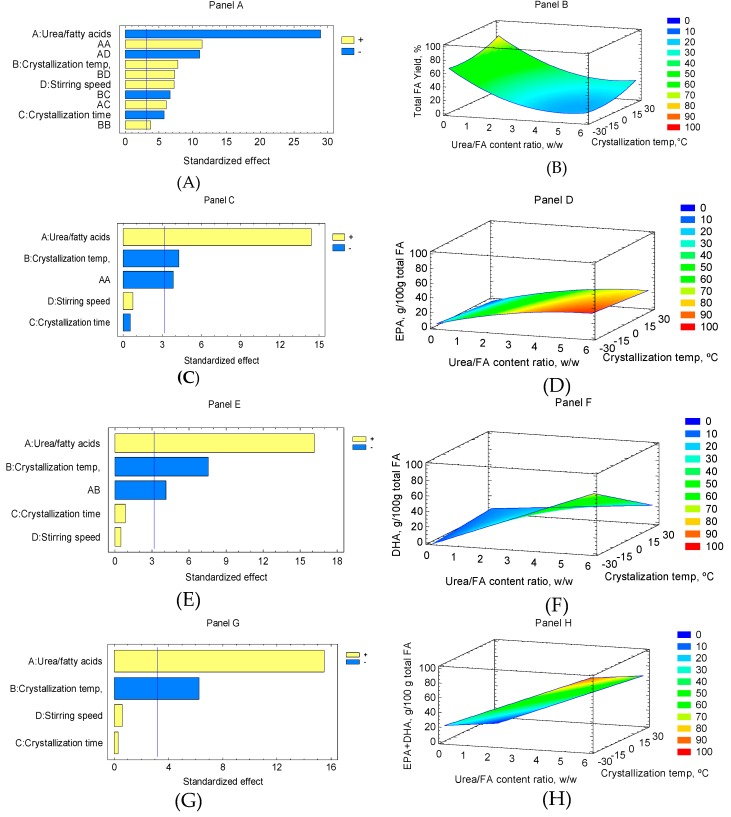
Pareto charts and response surfaces for the effects of different process variables: Panels in total FA yield (%, panels **A**,**B**), EPA content (g/100 g total FA, panels **C**,**D**), DHA content (g/100 g total FA, panels **E**,**F**), EPA+DHA content (g/100 g total FA, panels **G**,**H**). A: urea/FA contents ratio, *w*/*w*; B: crystallization temperature, °C; C: crystallization time, h; and D: stirring speed, rpm).

Pareto charts (Figure 1) were obtained for the different dependent variables as a function of the concentrate processing variables from RCSO; furthermore, the linear, quadratic, and interaction terms in the second-order polynomial were used to generate a three-dimensional response surface graph. Panel (A) indicates that the total FA yield (R1) of concentrates was dependent (*p* < 0.05) on the linear terms of the urea:FA content ratio (A), crystallization temperature (B), crystallization time (C), stirring speed (D), the quadratic terms AA and BB, and the interactions terms AD, BD, BC, and AC. Figure 1 (Panel B) shows the response surface of the urea complexation process for the total FA yield. The total FA yield decreased when the urea:FA content ratio increased and the crystallization temperature decreased. A similar result was found for the total FA yield effect in concentrate obtained from a by-product of rainbow trout processing where the total FA yield presented a minimum value in the response surface analysis when considering high urea:FA content ratios, at low crystallization temperature levels and stirring speeds, and at intermediate levels of crystallization time [17]. In the case of the EPA content (Figure 1, Panel C), the Pareto charts showed that certain linear terms—the urea:FA content ratio (A), crystallization temperature (B), and the quadratic urea:FA content ratio (AA)—provided a significant effect (*p* < 0.05). However, linear terms such as the crystallization time (C) and stirring speed (D) did not produce significant changes (*p* > 0.05). Figure 1 (Panel D) exhibits the response surface of the urea complexation process for EPA content. It was found that the EPA content increased with the urea:FA content ratio while it decreased as the crystallization temperature increased. For the DHA content (Figure 1, Panel E), the Pareto charts reported that the linear terms of the urea:FA content ratio (A), crystallization temperature (B), and the interaction between the urea:FA content ratio and the crystallization temperature (AB) revealed a significant effect (*p* < 0.05). The DHA content increased when the urea:FA content ratio increased, while it decreased as the crystallization temperature increased (Figure 1, Panel F). Finally, concerning the EPA+DHA content, the urea:FA content ratio showed a positive effect, whereas the crystallization temperature had a negative effect (*p* < 0.05) (Figure 1, Panel G). These results agree with those obtained by authors concerning the employment of the Asian catfish (*Pangasius bocourti*) and by-product of rainbow trout oil, which reported an inverse relationship between the urea:FA content ratio and the crystallization temperature on the urea concentrate process [16,17]. When the urea:FA content ratio increased and the crystallization temperature decreased as result, high values were obtained for DHA in the non-urea complexing fraction, as well as a great retention of saturated and monounsaturated FA in the urea crystal adducts. In this case, similar results for the EPA content were found. In all the cases, the EPA, DHA, and EPA+DHA contents did not significantly varied depending on the stirring speed (*p* > 0.05).

#### 2.2.3. Models Obtained for the Concentration of EPA, DHA, and EPA+DHA

Equations obtained for the experimental process variables of the response surface model calculated by multiple regression are shown in Table 2. According to the equations obtained, all the response variables were found to be dependent on the same process variables expressed in the Pareto analysis (Figure 1). The four RCSO concentrated models had an adjusted R^2^ by degrees of freedom of 72.0% for total FA yield (Equation (1), 84.0% for EPA (Equation (2)), 81.0% for DHA (Equation (3)), and 80.0% for EPA+DHA (Equation (4)). Such values reported that the models adequately represented the variability of the results. Since the *p*-values obtained for lack-of-fit in the ANOVA study (0.014, 0.35, 0.19, 0.28; Equation (1) to Equation (4), respectively) was greater or equal to 0.05, except for Equation (1), the model appears to be adequate for the observed data at the 95.0% confidence level.

#### 2.2.4. Independent Variables and Multiple Response Optimization

Table 3 (part a) shows the optimization of the independent variables for the response variables (EPA, DHA, and EPA+DHA) of the urea complexation process. The optimum values of dependent variables for EPA, DHA, and EPA+DHA content were 33.01, 76.81, and 98.85 (g/100 g total FA), respectively. In all the cases, a tendency toward the same values for the urea:FA content ratio and crystallization temperature was observed, so that the optimum EPA, DHA, and EPA+DHA contents were obtained.

Table 3b shows the levels of factors that maximized the EPA, DHA, and EPA+DHA contents (g/100 g total FA) by means of multiple response optimization and the stationary point that predicted a maximum of 30.71, 62.94, and 90.07 (g/100 g total FA) in EPA, DHA, and EPA+DHA, respectively. Maximum desirability score of 1 (range, 0–1) was attained. A maximum predicted value could be obtained, provided that the following process conditions were applied: 5.84 (urea: FA content ratio), −17.69 °C (crystallization temperature), 14.83 h (crystallization time), and 453.00 rpm (stirring speed).

**Figure 2:**
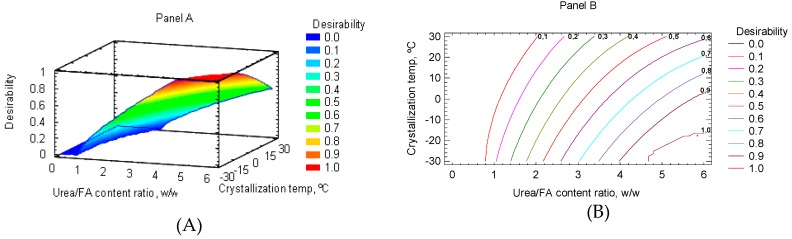
Combination of factors to maximize the desirability function for RCSO concentrate: response surface (**A**) and contour surface (**B**).

Figure 2 (panels A, B) shows the contours and estimated response surface of the urea:FA content ratio and crystallization temperature of the combination of factors levels to maximize the desirability function for RCSO concentrate. It is observed that the highest desirability values were reached by taking into account the high values of the urea:FA content ratio and the low crystallization temperature values. These results were similar to those previously reported for rainbow trout belly oil [17], where a maximum desirability score of 0.91 was obtained in the multiple response optimization of EPA, DHA and EPA+DHA contents. Furthermore, the predictive values of 32.50, 37.00, and 67.70 (g/100 g total FA) in the by-product concentrate of rainbow trout oil, respectively, were obtained. As a result, the following process conditions were applied in such study: 4.21 (urea:FA contents ratio), −15.00 °C (crystallization temperature), 24.0 h (crystallization time), and 1000 rpm (stirring speed).

#### 2.2.5. Validation of the Optimized Process and Characterization of the EPA+DHA Content Obtained

Table 3c shows the validation of multiple response optimization after experimentally performing the process conditions. For EPA content, comparison of the predicted value and the value obtained experimentally showed that both values were similar (i.e., 30.71 and 31.20 g/100 g total FA, respectively); however, the experimental value of DHA content was substantially different from the predictive value (49.31 and 62.94 g/100 g total FA, respectively). Experimental values performed on rainbow trout belly oil [17] were shown to be similar to the predicted values obtained in the current study for EPA and DHA (36.10 and 47.70 g/100 g of total FA, respectively). In the experimental validation, an 80.51 g/100 g total FA value was obtained for the EPA+DHA content, which agrees with the value revealed by other authors who obtained a stationary point of 89.38% for the EPA+DHA content [14].

### 2.3. Composition of FA in the RCSO Optimized Concentrate after Validation

Table 1 shows the FA composition of the RCSO compared to the composition of FA from the validated optimum concentrate. Optimization process validation was performed experimentally by combining the factors levels in which the optimal EPA+DHA content was attained (Table 3b,c). In these experiments, the effect of the urea complexation process on the composition of FA and FA groups in the optimized concentrate (g/100 g FA) after validation can be observed. When compared to the initial RCSO, there was a marked increase in the concentration of total PUFAs (96.99%) and a substantial decrease in the concentration of SFAs (0.59%) and MUFAs (2.42%). Furthermore, the predominant FAs in the optimum concentrate oil were EPA, DHA, and EPA+DHA (31.20, 49.31, and 80.51 g/100 g total FA, respectively). The urea complexation under the optimization process conditions has revealed a high efficiency, since a marked increase of total PUFAs from 38.24 to 96.99 g/100 g total FA could be observed; this increase was 1.21 times higher than that reported by Pando et al. [18]. Additionally, the total EPA+DHA final content was 2.5 times higher than that reported for refined salmon oil concentrate without the optimization process [18].

## 3. Discussion

In the present research, the distribution of SFAs in RSCO showed that this FA group was mainly composed of palmitic, stearic, and myristic acids. Similar values have been established for the SFAs group from crude commercial salmon oil [18]; interestingly, refined commercial salmon oil and Asian catfish oil also showed these three FA as the major ones, but a higher presence of stearic acid compared with myristic acid was detected in our study [16,18]. Among the MUFAs, the most abundant in the present study were 18:1 9c, 16:1 9c, 18:1 11c, and 20:1 11c. The total n-3 long chain polyunsaturated fatty acids (*n*-3 PUFAs) content was 1.14 times higher than the content of total *n*-6 PUFAs, showing values higher than those previously reported in different kinds of oils obtained crude commercial salmon, and rainbow trout (*Oncorhynchus mykiss*) [18,20]. The analysis of process variables shows a high recovery performance of RCSO concentrate when the urea:FA content ratio has the lowest value, and the value considered for the crystallization temperature is the highest. The results show a higher content of EPA and DHA when the total FA yield is lower, indicating that this experiment eliminated most of the SFAs and MUFAs from the starting oil, only leaving a small fraction of such acids in the urea non-complexed fraction. Similar values have been observed by other authors [17,21], who reported major yields with higher stirring speed, corroborating a significant effect of the stirring speed on the total FA yield in the urea complexation process (*p* < 0.05). Current results show an inverse relationship between the urea:FA content ratio and the crystallization temperature on the DHA content, which is a conclusion that has already been than those reported by several authors, whose employed different oils from marine origin such as seal blubber oil, tuna oil, Asian catfish oil, and a by-product of rainbow trout [13,14,16,17]. In this case, results similar for EPA content were found. As an explanation for this, it could be argued that the urea:FA content ratio has a significant positive effect (*p* < 0.05) on the concentration of EPA and DHA, probably as a result of increasing the concentration of urea with respect to that of FFA; consequently, this would lead to an increase of the number of adducts formed between the flat structures of SFA and urea molecules, this favoring the formation of hexagonal complexes and crystals [13], and leading to an increased concentration of EPA and DHA in the non-urea complexing solution. Previous studies on tuna oil concentrates have revealed that the regression models for total FA yield and total EPA+DHA content were highly significant with satisfactory R^2^ coefficients [14,22]. The present data proved that the urea complexation under the optimization process conditions has shown to be highly efficient, since an increase in the total PUFAs content from 38.24 to 96.99 g/100 g total FA could be reached, which was 1.2 times higher than that reported by Pando et al. [18]. Additionally, the total EPA+DHA final content was 2.5 times higher than that reported for refined salmon oil concentrate without applying an optimization process [18]. On the other hand, the oxidative and hydrolytic stability parameters (PV, pAV, TOTOX, and FFA) indicate that the quality of the oil used in this study was included within the acceptable quality limits for edible fats and oils of marine origin according to the Chilean Food Sanitary Regulations. Government institutions and trade associations, such as the Council for Responsible Nutrition (CRN) and the Global Organization for EPA and DHA Omega-3s (GOED), have set strict guidelines for marine oil quality and safety parameters. According to the previously described recommended values [23,24], the starting RCSO can be considered included in the 98% of fish oil products compliant with the PV limit of 10 meq. active oxygen kg^−1^ oil set by British Pharmacopeia Fish Oil Type I, European Union (EU) Pharmacopeia Fish Oil Type I, and Australian government guidelines. Furthermore, salmon oil samples were compliant with the pAV limit of 15 set by British and EU Pharmacopeia Fish Oil Type II, as well as the pAV limit of 20 set by the GOED, Canada Natural Health Product Directorate (NHP), United States Pharmacopeia, and Codex Alimentarius Commission, Food and Agriculture Organization of the United Nations (CODEX/FAO). Thus, the primary and secondary lipid oxidation levels of RCSO are not exceeding the regulatory thresholds in the testing peroxide values and p-anisidine values. Interestingly, such lipid oxidation scores can be considered similar to those previously obtained for refined salmon oil samples [18] and salmon oil [24]. On the other hand, the FA composition of the RCSO showed that most abundant FAs were C18:1 9c, 18:2 9c, 12c, and C16:0, followed by EPA and DHA. The value of the total SFAs was found to be 21.28% in RCSO. Concerning PUFAs, the most abundant in RCSO concentrates were DHA, EPA, and 18:2 9c, 12c.

## 4. Materials and Methods

### 4.1. Materials and Chemicals

Refined commercial salmon oil was provided by Fiordo Austral S.A. (Puerto Montt, Chile). Fatty acid methyl ester (FAME) standards, fatty acid (FA) standards, and C23:0-methyl ester (2COT N-23M-A29-4 NU-CHECK-PREP-INC) were obtained from NU-CHECK-PREP, INC (Elysian, MN, USA). All the solvents and chemicals used (including urea, ethanol, α-tocopherol, and *n*-hexane) were of analytical grade (Merck, Santiago, Chile).

### 4.2. Characterization of Refined Commercial Salmon Oil

Initial RCSO characterization was carried out by chemical analyses. For it, the following standard Association of Official Analytical Chemists (AOAC) and official methods [25] were carried out: peroxide value (PV; method Cd 8b-90:1-2), p-anisidine value (pAV; method Cd 18-19:1-2), and total oxidation value (TOTOX; method Cg 3-91) and free fatty acids (FFA) contents (method Ca 5a-40:1).

### 4.3. FA Composition of the RCSO and n-3 LCPUFA Concentrates

For analyzing the FA composition of the RCSO and the different LCPUFA concentrates, a methylation process was performed to obtain FAMEs. For it, a two-step process was performed, according to previous research [18,26]. FAME analysis was carried out on an HP 5890 series II GLC with a flame ionization detector (FID) with the injection system split. A fused silica capillary column (100 m length × 0.25 mm × 0.2 µm film thickness) coated with SPTM-2560 (Supelco, Bellefonte, PA, USA) was used [18,26,27,28,29]. DataApex ClarityTM software (DataApex Ltd., Prague, Czech Republic) for chromatogram analysis was applied. The reference standard NU-CHEK GLC463 was used to identify the FA profiles. The concentration of the different FAME was determined from the calibration curves by assessment of the peak/area ratio. The quantification of all the individual FAs (g/100 g total FA) was achieved by employing C23:0 methyl ester as the internal standard according to the (AOCS Official Method (Ce 1j-7, 2009) [26].

### 4.4. n-3 LCPUFA Concentrates from RCSO

The procedure included salmon oil saponification, which was in agreement with other authors [17,18,30]. Concentrate from RCSO was prepared by FFA collection, the formation of urea FFA inclusion complexes, and the extraction of free n-3 LCPUFA. For it, the urea complexation method was carried out by 28 experimental runs with different urea:FA content ratios (0 to 6 *w*/*w*), crystallization temperatures (−30 to 30 °C), crystallization times (0 to 48 h), and stirring speeds (0 to 800 rpm). The FFAs were mixed with urea and 95 % ethanol, and the mixture was subsequently stirred and heated at 60 °C with magnetic stirring. Then, it was cooled with constant stirring to different conditions of temperature and time as described in the experimental design. The crystals formed were separated from the liquid phase by filtration with a Whatman No.1 paper. The non-urea complexing fraction was diluted with 100 mL of distilled water by each 10 g, acidified to pH 4.5 with 6 N HCl, and washed in a separating funnel with hexane (400 mL). The hexane phase was filtered with anhydrous sodium sulfate in a Whatman No.1 paper, and the solvent was partially removed using a rotatory evaporator at 40 °C under vacuum [16,17]. The resulting n-3 LCPUFA concentrates were stored at −80 °C with 0.5% of α-tocopherol under nitrogen atmosphere until use for further analysis.

### 4.5. Experimental Design and Optimization Procedure

A rotational central composite design 2^4^ + star, with 4 factors and 5 levels, was carried out, with 28 experimental runs that included 4 repetitions of the central point based on the RSM. The following conditions for the independent variables were considered (Table 1): urea:FA content ratio (variable A: 0 to 6 *w*/*w*), crystallization temperature (variable B: −30 to 30 °C), crystallization time (variable C: 3.05 to 48.0 h), and stirring speed (variable D: 0 to 800 rpm). On the basis of the non-urea complexing fraction, the following response variables (R variables) of the experiment design were chosen: total FA yield (variable R1: g FA in the non-urea complexing fraction/100 g initial RCSO), EPA content (variable R2: g/100 g total FA in concentrate), and DHA content (variable R3: g/100 g total FA in concentrate). Four replicates were carried out at the central point of the experimental design in order to evaluate the experimental error. All experiments were performed randomly to minimize the effect of unexplained variability in responses resulting from extraneous factors [31]. In order to obtain response surfaces, multiple regression equations were fitted to the responses obtained by discarding non-significant terms (*p* > 0.05) to obtain response surfaces. To maximize the desirability function a multiple response optimization was performed to optimize several responses simultaneously, which maximized the desirability function scores that ranged between 0 and 1 [32]. The RSM was used to optimize and maximize the response variables, and a quadratic polynomial regression model was assumed for predicting individual Y variables. The model proposed for each Y value was as according to Equation (5):

(5) $$Y_{i}=\beta_{0}+\sum_{i=1}^{4}\beta_{i}X_{i}+\sum_{i=1}^{4}\beta_{ii}X_{i}^{2}+\sum_{i=1}^{3}\sum_{j=i+1}^{4}\beta_{ij}X_{i}X_{j}+\varepsilon ,$$

where β_0_, β_i_, and β_ii_ represent the intercept, linear, and quadratic coefficients, respectively; β_ij_ corresponds to the interaction coefficient terms for the interaction of variables i and j; X_i_ represents the independent variables; and ɛ denotes to the random error [31,32].

### 4.6. Statistical Analysis

Multiple regression analysis, ANOVA. canonical, and ridge maximum of data in the response surface regression (RSREG) procedure was used. The estimated response surface and contours of the estimated response surface were developed using the fitted quadratic polynomial equations obtained from the response surface regression (RSREG) analysis, holding the independent variables with the least effect on the response at a constant value and changing the levels of the other two variables [31,32]. A multiple-response optimization was performed to assess the combination of experimental factors that simultaneously optimize several responses; as a result, maximization of the desirability function was obtained, this function ranging from 0 to 1 [32]. Analyses were performed in triplicate considering the standard deviation of each sample. The lack-of-fit test was carried out by comparison of the variability of the current model residuals with the variability between observations at replicate settings of the factors [31,32]. Statgraphics Centurion XV.II (Manugistics Inc., Rockville, USA) was used.

## 5. Conclusions

The physical–chemical analyses of refined commercial salmon oil indicate that it is a good quality raw material that complies with the characteristics that are typical of oils of marine origin. A high concentration of EPA and DHA was obtained from RCSO, achieving an increase of up to 4.1 and 7.9 times in the concentrate, with values of 31.20 and 49.31 g/100 g total FA, respectively. Interestingly a 5.8-time increase was observed for the EPA+DHA content, from 13.78 to 80.51 g/100 g total FA. Therefore, it has been proved that it is possible to maximize the EPA and/or DHA content by the optimization of the variables in the urea inclusion process. The results of this study could serve as a basis for the food and nutraceutical industry, whose processes with clean technologies can develop new functional foods enriched with EPA and DHA. Furthermore, the consumption of such new functional foods would be likely to produce a positive and profitable impact on the health of a wide range of consumers such as pregnant women, infants, and older adults in general.

## Figures and Tables

**Table 1:** Composition of fatty acids in (RCSO) and the optimized concentrate from RCSO (g/100 g total FA) *.

FA or FA Groups	RCSO	RCSO Optimum
12:0	0.07	Nd
14:0	3.19	0.12
15:0	0.20	0.09
16:0	13.74	Nd
16:1 9t	0.15	Nd
16:1 7c	Nd	Nd
16:1 9c	4.66	0.51
16:1 11c	Nd	Nd
16:1 13c	Nd	Nd
17:0	0.13	0.10
17:1 10c	0.56	1.28
18:0	3.69	0.28
18:1 9c	29.61	0.59
18:1 11c	3.69	0.04
18:2 9t, 12t	Nd	Nd
18:2 9c, 12c	16.69	7.48
18:2 9c, 15c	Nd	Nd
18:3 6c, 9c, 12c	0.22	1.09
20:0	0.26	Nd
18:3 9c, 12c, 15c	3.25	2.60
20:1 5c	Nd	Nd
20:1 8c	Nd	Nd
20:1 11c	1.60	Nd
18:4 6c, 9c, 12c, 15c	Nd	Nd
20:2 11c, 14c	0.79	0.05
20:3 8c, 11c, 14c	0.30	1.16
20:3 11c, 14c, 17c	0.12	0.03
20:4 8c, 11c, 14c, 17c	0.40	1.37
22:1 13c	0.21	Nd
20:5 5c, 8c, 11c, 14c, 17c	7.53	31.20
24:1 15c	Nd	Nd
22:5 7c, 10c, 13c, 16c, 19c	2.69	2.70
22:6 4c, 7c, 10c, 13c, 16c, 19c	6.25	49.31
Total SFAs	21.28	0.59
Total MUFAs	40.48	2.42
Total PUFAs	38.24	96.99
Total *n*-3PUFAs	20.45	87.21
Total *n*-3LCPUFAs	18.08	85.82
EPA+DHA	13.78	80.51

* Abbreviations employed: DHA (docosahexaenoic acid), EPA (eicosapentaenoic acid), FA (fatty acid), SFAs (saturated fatty acids), MUFAs (monounsaturated fatty acids), RCSO (refined commercial salmon oil), n-3LCPUFAs (n-3 long chain polyunsaturated fatty acids), Nd (not detected).

**Table 2:** Values obtained for the experimental and predicted response variables of RCSO concentrate by central composite rotatable design 2^4^ + star based on the response surface methodology ^1^.

Run	Process Variables ^*^				Response Variables ^**^					
					Experimental Values			Predicted Values		
	A	B	C	D	R_1_	R_2_	R_3_	R_1_’	R_2_’	R_3_’
1	1.5	−15	14.3	200	40.44	10.47	11.04	33.77	13.77	14.00
2	4.5	−15	14.3	200	12.64	28.41	44.38	12.23	28.09	45.89
3	1.5	15	14.3	200	48.71	9.22	9.52	40.35	9.52	10.23
4	4.5	15	14.3	200	17.45	25.09	32.69	18.82	23.85	26.94
5	1.5	−15	36.8	200	36.25	10.46	11.13	29.02	13.22	15.24
6	4.5	−15	36.8	200	10.44	24.42	55.91	21.55	27.55	47.14
7	1.5	15	36.8	200	4.22	7.96	8.18	20.22	8.98	11.47
8	4.5	15	36.8	200	18.12	24.87	30.47	12.76	23.30	28.18
9	1.5	−15	14.3	600	41.74	10.23	10.89	44.89	14.52	14.71
10	4.5	−15	14.3	600	12.77	30.20	46.43	12.14	28.84	46.61
11	1.5	15	14.3	600	78.45	9.02	9.41	68.43	10.27	10.94
12	4.5	15	14.3	600	16.11	25.16	34.34	21.40	24.60	27.65
13	1.5	−15	36.8	600	45.49	9.91	10.06	40.14	13.97	15.96
14	4.5	−15	36.8	600	9.86	27.56	48.55	7.17	28.30	47.85
15	1.5	15	36.8	600	58.94	7.84	7.91	48.30	9.73	12.18
16	4.5	15	36.8	600	12.64	23.05	28.93	15.33	24.05	28.89
17	0	0	25.5	400	57.38	6.33	6.39	67.84	-0.77	0.94
18	6	0	25.5	400	15.86	25.45	30.43	13.34	27.88	49.55
19	3	−30	25.5	400	12.21	29.26	44.37	19.61	24.94	36.61
20	3	30	25.5	400	33.82	13.52	14.65	34.36	16.45	13.88
21	3	0	3.05	400	12.71	20.41	22.67	25.65	21.24	24.00
22	3	0	48.0	400	16.31	23.01	28.93	14.83	20.15	26.49
23	3	0	25.5	0	19.11	22.62	25.72	13.39	19.95	24.53
24	3	0	25.5	800	16.47	26.07	33.39	27.09	21.44	25.95
25	3	0	25.5	400	20.77	22.43	25.82	20.24	20.69	25.24
26	3	0	25.5	400	20.74	21.40	25.20	20.24	20.69	25.24
27	3	0	25.5	400	17.20	24.00	29.15	20.24	20.69	25.24
28	3	0	25.5	400	22.74	18.23	20.23	20.24	20.69	25.24
**Equations**									**R^2^ Adjusted**	
**Total FA yield** = 57.60 − 19.47A + 0.26B − 0.87C + 0.08D + 2.26AA+ 0.01BB + 0.21AC − 0.02AD − 0.02BC + 0.001BD									0.72(1)	
**EPA** = −0.90 + 9.53A − 0.14B − 0.79AA									0.84(2)	
**DHA** = −1.19 + 8.10A + 0.17B − 0.19AB									0.81(3)	
**EPA+DHA** = 3.52 + 12.88A − 0.52B									0.80(4)	

^1^ Central composite design: 2^4^ + star, which studies the effects of 4 factors in 28 runs based on the RSM. * Independent variables: A (urea/FA content ratio, *w*/*w*), B (crystallization temperature, °C), C (crystallization time, h), and D (stirring speed, rpm).** Response variables: R_1_ (total FA yield, g FA in the non-urea complexing fraction/100 g initial saponified oil FA), R_2_ (EPA content, g/100 g total FA), and R_3_ (DHA content, g/100 g total FA). Predicted response variables: R_1_’, R_2_’, and R_3_’.

**Table 3:** Process variables * optimization and multiple response optimization of the response variables.

**Part a) Optimization of the Process Variables**						
**Dependent Variables**	**Process Variables**				**Stationary Point**	Optimum Value **
	A	B	C	D		
EPA	5.99	−29.79	3.05	599.00	Maximum	33.01
DHA	6.00	−29.98	48.05	108.30	Maximum	76.81
EPA+DHA	6.00	−29.95	47.79	271.36	Maximum	98.85
**Part b) Multiple Response Optimization of the Response Variables**						
**Dependent Variables**	**Process Variables**				**Stationary Point**	Predicted Value **
	A	B	C	D		
EPA	5.84	−17.69	14.83	453.36	Maximum	30.71-
DHA						62.94
EPA+DHA						90.07
Maximum desirability						1.0
**Part c) Experimental Validation of the Multiple Response Optimization of the Dependent Variables**						
**Dependent Variables**	**Process Variables**				**Stationary Point**	Experimental Value **
	A	B	C	D		
EPA	6.00	−18	14.80	500	Maximum	31.20
DHA						49.31
EPA+DHA						80.51

* Process variables (A, B, C, and D) as expressed in Table 2. ** Values expressed as g/100 g total FA.
